# Improving Functioning of Children Birth to Five with Emotional and Behavioral Problems: The Role of Comprehensive Mental Health Services and Supports

**DOI:** 10.3390/pediatric15010005

**Published:** 2023-01-09

**Authors:** Alison K. Cohen, Tracy Hazelton, Henrissa Bassey, Margie Gutierrez-Padilla, Carolyn Novosel, Chloe R. Nichols, Sonia Jain

**Affiliations:** 1 Department of Epidemiology & Biostatistics, School of Medicine, University of California, San Francisco, CA 94158, USA; 2 Alameda County Behavioral Health Care Services, Oakland, CA 94606, USA; 3 Data in Action, Oakland, CA 94611, USA; 4 Department of Health Sciences, Northeastern University, Boston, MA 02115, USA

**Keywords:** childhood, health promotion, health services, mental health, program evaluation, public services

## Abstract

Introduction: Emotional and behavioral problems are growing among children ages birth to five, and racial/ethnic and socioeconomic disparities exist. Comprehensive, culturally responsive, family-driven systems of care, such as the one operated by California’s diverse, urban Alameda County, offer one potential intervention. Methods: We used client-level service data (*n =* 496 children) to calculate descriptive statistics and regression analyses (including multilevel models to account for observations for the same client at multiple points in time). We estimated the prevalence of mental health issues and assessed the association between the length of time using services and emotional and behavioral functioning. Results: Comprehensive mental health services and supports were associated with improved emotional and behavioral functioning outcomes for children over time, even after controlling for other risk factors. Discussion: Systems of care appear to support the multidimensional functioning of children and their families.

## 1. Introduction

### 1.1. Prevalence and Implications of Children’s Mental Health Issues

Approximately 10% of children ages 0–5 in the United States of America (USA) experience social, emotional, and/or behavioral problems that hamper their functioning and development over their lives [1] and low-socioeconomic position children and BIPOC (Black, Indigenous, and People of Color) children disproportionately experience such problems [2]. 

In the USA, some local government agencies provide early childhood mental health services to low-income families on Medicaid, which is publicly provided healthcare insurance for individuals whose income is below or near the poverty line. However, their effectiveness in reducing emotional and behavioral problems is largely unexamined, especially for diverse communities and low-income children of color [3,4]. 

### 1.2. Determinants for Early Childhood Social-Emotional and Behavioral Problems

Multiple levels of factors affect social-emotional and behavioral problems in early childhood. Neighborhood factors, including food insecurity [5], housing and economic instability [6,7,8], and community violence [9], affect children’s social, emotional, and behavioral functioning and development. Family factors, including parental mental health [10], parental neglect and substance abuse [11,12], and exposure to violence at home [13] can affect behavioral issues in childhood and into adolescence and adulthood. There can also be health care system factors. For example, limited access to culturally competent health services can affect health service utilization, which can in turn affect health [14]. In general, increasing protective factors (e.g., social support, family support, early intervention) can promote better mental health [15]. Since child and family functioning issues can accumulate and persist [16], emotional and behavioral functioning in early childhood offers an opportunity for intervention [17].

### 1.3. Early Childhood Systems of Care

Several early intervention strategies exist, including team-based wraparound or integrated service provision, in which the delivery of comprehensive, strengths-based care is individualized [18] and mental health consultation [19], but few take a comprehensive, systems-level approach [20]. Given the complexity of overlapping issues that can present during early childhood, a comprehensive system of care (SOC) approach that provides multiple coordinated services within an integrated family-driven system is a promising solution.

SOCs are models of service organization and delivery [21] that simultaneously implement multiple approaches that are family-driven, data-driven, community-based, and comprised of practitioners who share a dedication to learning about others’ cultures in addition to examining their own beliefs and cultural identities, or are “culturally humble” [22,23]. The USA’s Substance Abuse and Mental Health Service Administration (SAMHSA) has funded developing comprehensive children’s SOCs (including early childhood SOCs) with the goal of improving child-level, family-level, clinic-level, and system-level mental health outcomes; SOCs can also be funded by other sources, including private funding. Grounded in socio-ecological theory [24]. These SOCs provide an integrated continuum of services and supports, including non-clinical services (e.g., educational, recreational, and social services), that work across multiple child-serving systems to collectively meet children’s needs [25,26].

Most children entering SOCs have clinically significant emotional and behavioral symptoms [1,27]. SOC approaches may improve children’s social and emotional functioning and development [1] and increase functioning [28], but few studies have used secondary data to examine the association between participating in SOCs over time and early childhood functioning. Those studies had mixed findings, including no significant change in children’s social and emotional competencies, and significant decreases in children’s internalizing and externalizing problem behaviors [29]. Our study leverages data to evaluate Alameda County’s early childhood SOC. Recent practice-based literature has highlighted implementation challenges SOCs have faced, with a particular focus on the importance of accommodations for culturally and geographically diverse populations [30]. Our study adds to this literature by examining the effectiveness of a SOC in a culturally diverse county.

### 1.4. Alameda County and Early Connections

Alameda County is a large urban county located in the San Francisco Bay Area’s East Bay [31]. California’s racial/ethnic diversity is often identified as a harbinger of the U.S.’s future demographics [32], and Alameda County is even more diverse than the rest of California [33]. 

Early Connections, Alameda County’s early childhood initiative that was supported in part by SAMHSA starting in 2009, sought to strengthen the existing early childhood SOC and shift towards a strengths-based approach to family-driven care [34]; more details at https://sites.google.com/site/acearlyconnect/home (accessed on 15 October 2022). This initiative worked with 14 existing community-based mental health providers to serve low-income families on MediCal (California’s Medicaid). Early Connections emphasized linking parents and providers as equal partners in caring for children and engaging local agencies in this effort [34]. 

Early Connections served children aged 0–5 with serious social, emotional, and/or behavioral concerns, including those in subsidized child care programs, Early Head Start and Head Start, those who received primary medical care through California Child Health and Disability Prevention medical providers, and those in the child welfare system. By targeting these vulnerable populations, the countywide initiative attempted to minimize racial, cultural and socioeconomic disparities in access to care observed elsewhere [35]. 

Early Connections emphasized being family-driven and culturally responsive [33], and worked to build workforce capacity to increase commitment to cultural humility and co-learning [34]. Early Connections also studied the current experiences of families from multiple cultures to inform improvements [33,36]. It offered a SOC that sought to mitigate existing fragmentation to better support children with serious mental health needs by integrating several providers and agencies to make them more easily accessible to children and their families. This included integrating Early Periodic Screening, Diagnosis and Treatment (EPSDT) mental health services (federally required for MediCal-insured children); MediCal (California’s Medicaid); the state- and county-administered Child Health and Disability Prevention services; California Children’s Services; school districts’ Early Childhood Special Education services; Regional Centers that support children with developmental disabilities; Early Head Start and Head Start; and local hospitals, child welfare, public health, family-run organizations and other child-serving institutions. 

We posit that lessons can be learned from this local model because it operationalizes multiple innovative promising models from the field, including SOC and family-driven services, and because the core principles of SOC emphasize the importance of locally guided work [26]. Additionally, learning from regional variations in implementing SOCs could lead to the overall improvement in services provided as communities learn from each other. 

### 1.5. Current Study

We use client-level service data collected by Alameda County Behavioral Health Care Services via mental health providers to assess the effectiveness of continued participation in a county-wide Mental Health Services and Support system on emotional and behavioral functioning outcomes of high-risk children. Informed by Yin’s approach to case studies [37], this case is revelatory because we studied an innovative phenomenon within a relevant, diverse setting. We examined the effect of mental health services—in particular, the SOC approach to service provision—on the emotional and behavioral functioning of a diverse, longitudinal sample of children birth-5, accounting for covariates. We hypothesized that participation in Alameda County mental health services would be associated with improved emotional and behavioral functioning. 

## 2. Methods

### 2.1. Data Sources and Measures

We present secondary data analyses of two merged client-level datasets based on two data collection tools: The Community Functioning Evaluation (CFE) Form birth-5 Version (CFE 0–5) and The Community Registration Form. The CFE form includes mental health data compiled by the Alameda County Department of Behavioral Health Care Services (ACBHCS) and collected in the 2010 calendar year by the 14 EPSDT providers in the County on each child enrolled in services during that time period. This was the only time range of data shared with the researchers. ACBHCS spent over a year developing a community-friendly form in partnership with providers and family members; this process led to the selection of each of the items included in the form, and therefore did not rely exclusively on prior psychometric properties. A clinician was encouraged to complete this form on every new child enrolled for services in the county, and again every 6 months and at discharge, providing client-level longitudinal data. This timeframe was not always achieved perfectly. The number of times the form was completed is our indicator of continued use of mental health services; all mental health services were part of the SOC. The second dataset, the Community Registration form, has demographics, including language spoken, socioeconomic status, insurance variables, race/ethnicity, gender, and age.

The 48-item self-administered CFE form asked parents to report child-level and family-level functioning. The CFE team a priori organized the items into 8 domains: early care and education, emotional and behavioral functioning (EBF), developmental functioning, health, basic needs, safety, social relationships, and family functioning, each of which was assessed using Cronbach’s alpha to confirm psychometric validity (Table 1). For each of the eight domains, we summed items to create a summary score, with a value of 0 assigned to each item that was described as “no problem,” and 1 for items that were described as problems. 

This research was approved by the WestEd Institutional Review Board. 

### 2.2. Sample

The data include all children aged birth to five receiving MediCal mental health services in 2010 in Alameda County. These data were shared with the authors in 2011. For descriptive statistics about the population, the 496 children who had at least one recorded observation in 2010 were analyzed. For the longitudinal analyses, we analyzed data from the subsample of children who had multiple CFE 0–5 forms recorded (*n =* 148); in this sample, the average time between their first and second CFE form was 102 days, and the average time between their second and third CFE forms (for the 46 children who had three or more forms) was 98 days. In both the overall population and the longitudinal subsample, participants came from the same 14 different clinics.

Due to limitations of the administrative data, we do not know whether the first intake form in 2010 was the child’s first intake form in the system (i.e., a new enrollee) or not. Therefore, while we can look at change over time, we are not necessarily documenting the change over time between intake and subsequent follow-ups.

### 2.3. Data Analysis

We calculated descriptive statistics and conducted multilevel linear regressions (to control for clustering by clinic) in the sample of unique clients (*n =* 496) to describe the population using mental health services and their needs, and explore links between child and family characteristics and child emotional and behavioral functioning. 

Then, we assessed if the time spent receiving services, within the context of a nascent SOC approach, was associated with improved outcomes. We conducted longitudinal analyses using repeated-measures random effects maximum likelihood estimation using the subsample of clients who had 2–3 follow-ups in the 2010 calendar year (*n =* 148, for whom we have 313 observations) as a crude measure of the length of Mental Health Services and Supports care per child. Four individuals had more than three data points; as this frequency of data collection was the outlier and potentially reflective more of the individual’s case history than the potential effect of mental health services and supports, and since it would contribute to a highly unbalanced design, only the first three 2010 follow-ups of these individuals were retained for analyses. Data were analyzed in Stata 11.2 (College Station, TX, USA).

## 3. Results

### 3.1. Sample Characteristics

In 2010, 14 clinics in Alameda County conducted 675 community functioning evaluations of 496 unique clients ages 0–5. These clients were disproportionately male (57.6%) (Table 2). Approximately 46.8% of children enrolled were Latinx, 25.2% Black, 13.3% White, 9.5% Asian/Asian American/Pacific Islander, 0.4% Native American, and 6% more than one race or other/unknown race. Over one-third (36.5%) spoke Spanish at home and 5.6% spoke other languages (mostly Asian). Black and Latinx children were overrepresented compared to the county’s birth to 5 resident population and nationally (Latinx: 24%, Black: 12%; [38]); and Whites and Asian Americans/Pacific Islanders were underrepresented compared to the county’s population and nationally (White: 52%, Asian: 5%; [38]). 

### 3.2. Percent Receiving Mental Health Services

There were multiple provider follow-ups for each client after enrollment into services. Of the 496 clients enrolled at baseline, 70.1% were receiving mental health services at 6 months, 22.8% at about 1 year, 6.3% had a CFE form completed at least three times, and 0.8% had four or more completed CFE forms. There were no statistically significant differences by age, gender, race/ethnicity, or language in the frequency of receipt of mental health services. 

### 3.3. Prevalence of and Associations between the Eight Domains Measured

At the first documented visit in 2010, children presented with multiple issues (Table 1). Almost all (97.3%) had at least one emotional or behavioral functioning issue—the reason for which they were referred for care originally. (The remaining 2.7% may have other reasons for referral, missing data, and/or improvement from their original intake.) Additionally, 83.7% had issues in five or more of the eight domains. Anxiety/fears (78%), separation problems (58%) and depression (45%) were the most common emotional issues. The ability to regulate (82%) and impulse control (76%) were the most common behavioral functioning issues.

### 3.4. Other Needs

A wide variety of other issues were quite common among those receiving services. Almost all reported family functioning issues (95.7%) and social relationship issues (89.2%). A majority (56.8%) also had exposure to violence, neglect, or abuse. Approximately 40% did not have their basic needs (e.g., housing, transportation, food) met. Approximately one-quarter (27%) had other health issues affecting their development (e.g., medical, dental, vision). 

In bivariate analyses, basic needs and safety were each associated (*p <* 0.05) with emotional and behavioral functioning, but when included in a multivariable linear regression model that also included family issues, they were no longer statistically significant (*p >* 0.2). In contrast, family issues (which included an item on parent/caregiver mental health) remained significantly associated with emotional and behavioral functioning (β = 0.46, *p =* 0.001). Correlations between each domain are depicted in Appendix A Table A1. 

### 3.5. Longitudinal Association between Mental Health Services and Child Functioning

Repeated check-in assessments (as a proxy for continued mental health services and supports) were associated with improved emotional and behavioral functioning outcomes, in both a bivariate analysis and also when controlling for age, race/ethnicity, and language spoken at home. Our random-effects multivariable regression analysis (Table 3) showed an average decrease in EBF score of 1.68 (95% CI: 0.88, 2.49) from the initial intake to the second follow-up, and an average decrease of 2.97 (95% CI: 1.51, 4.42) from the first intake to the third follow-up; all of these findings are statistically significant (*p <* 0.0005). Each additional completed CFE form (which corresponds to ~3 months of mental health services) was associated with a statistically significant, substantial decrease in average EBF score (Wald test chi-square statistic = 25.2 with 2 degrees of freedom; *p <* 0.00005). As a point of reference, the baseline mean EBF score, among the subsample of clients (*n =* 148) who had more than one data point in 2010 and controlling for age, race/ethnicity, and language spoken at home, was 8.25 (95% CI: 5.76, 10.75).

## 4. Discussion

The results are a promising use of administrative data to explore a program’s impact with no additional data collection burden to program staff. Our multilevel, multivariable longitudinal analysis of a regional sample found that participants’ continued use of Medicaid services provided through the Alameda County early childhood mental health system was associated with improved child emotional and behavioral functioning. This suggests that Alameda County’s early childhood mental health SOC may help improve emotional and behavioral functioning among high-risk children. Given that the study population was highly racially/ethnically diverse and all low-income, these findings have important implications for health equity. Additionally, Alameda County’s culturally responsive approach to mental health services offers a model for other counties that serve similarly diverse populations. In concert with growing evidence supporting integrated mental health services and supports and family-engaged continua of care [39,40,41], our findings support the continued growth of SOCs as a promising model for service provision. We also emphasize the importance of collecting longitudinal administrative data. 

### 4.1. Other Needs

We highlight the breadth of other needs affecting low-income young children with social and emotional concerns, including additional health issues and unmet basic needs. The prevalence is striking: 84% of children had issues in over half of the domains measured, and 41% had at least one basic need unmet. These are likely underestimates, given the complexity of the question (asking caretakers to assess if the problem existed and if it was severe enough to potentially affect the child’s development), as well as social desirability bias potentially leading caretakers to under-report problems. Since unmet basic needs are associated with increased social–emotional problems [5,6], we recommend future researchers explore how SOCs can address basic needs to promote child wellbeing. 

Our findings align with other studies documenting extensive additional needs among children with social-emotional and behavioral health issues [1,19,33,36,42]. Our quantitative findings also complement qualitative findings from focus groups of families conducted in the same population, which found that families valued integrated mental health systems and services to support their children’s mental health needs and that SOCs improved access to services and supports, parent-provider communication, and feelings of dedication to children’s development [33,36]. 

### 4.2. Mental Health Disparities

This study had a diverse sample. Latinx and Black children were disproportionately represented in the client population, illustrating the perniciousness of health disparities documented elsewhere [43]. Given the prevalence of family and community risk factors documented (e.g., poverty, high exposure to violence and trauma at home and in the community, parental substance abuse, and mental health issues), we recommend that mental health providers collect comprehensive information about such family and community conditions in a culturally humble way [23], and use these data to inform and improve children’s diverse environments. Additionally, there was a higher proportion of boys in our sample than girls. Although boys were disproportionately represented in comparison to Alameda County’s demographics, young male children are significantly more likely to exhibit symptoms of social, emotional, or behavioral issues than females [38]. This suggests the need for support which is attuned to the different experiences young boys and girls have in early childhood. 

### 4.3. Study Strengths and Limitations

We highlight our approach of using existing administrative data for program evaluation as a non-resource-intensive way to hone our understanding of early childhood. However, we did not have data regarding service type(s) or amount(s), so could not explore what services or levels of services were particularly effective. 

Since these are observational data, these results could also be explained by unmeasured confounding, including potentially heightened and/or over-reported symptomology at baseline, since all children had to have a DSM diagnosis to receive services. However, since some of these children had been enrolled in the program in 2009, their first data collection point in 2010 would measure their status after having begun to receive mental health services and support, which helps address this potential bias. (On the flip side, this means that we are only able to examine change over time, not change from initial intake.) 

Any longitudinal sample can be subject to selection bias. There could be many reasons for loss to follow up over time. For example, families who did not perceive improvements after the initial data collection/enrollment may have been less interested in returning for subsequent services, or they may have viewed their children as having improved enough to no longer need services, or it may have been too difficult to receive services. We do not have data on why individuals dropped out of the sample.

Additionally, there are limitations to the measures. Since the forms were designed in partnership with providers and families, the measures reflected balancing an interest in high psychometric validity with being easy for providers to complete and not being too burdensome on families. This process allowed for face validity. The Cronbach’s alphas for most of the measures are in the range of acceptability but may not be as refined measures of constructs as longer instruments designed specifically for research purposes.

### 4.4. Implications for Research and Practice

Future research should explore the mechanisms by which an integrated, community-based, family-driven, culturally responsive SOC approach can be successful, and if there are specific strategies that are particularly effective in improving child and family-level outcomes. This could entail using multilevel models to understand children nested within families nested within clinics nested within systems to understand how the different levels interrelate. Ideally, this could include data from multiple systems of care across a country, and/or internationally. 

Future research should capture families’ and children’s well-being over time. For example, this could involve examining social, emotional, and behavioral functioning into adolescence and seeing how trajectories vary by access and use of mental health services, including interactions with multiple service systems. Better understanding of effective, culturally responsive mental health services for Latinx, Asian and Black children, given their unique risk and protective factors, is also critical. 

We encourage future researchers to conduct similar regional case studies to bolster the body of evidence investigating SOCs and to understand what place-specific variations in impact may arise. We also note that data in our study came from one year (2010), and that systems of care approaches may have continued to advance. We encourage replicating this study with data from more recent years, in addition to in other populations, to test the generalizability of these findings. We also recommend bolstering administrative data records so that such analyses could have more detailed records about the services each participant received. This could allow researchers to determine what components of a system of care may be most effective. 

Our study finds that receiving services within a comprehensive, family-driven system of care is associated with improved well-being among young children from low-socioeconomic positions in California, but these findings are likely relevant for other contexts as well, including communities beyond the US. Most research to date has been within the US, so it would be important to test and observe what family-driven systems of care could look like in other countries, including communities in the Global South.

## 5. Conclusions

This study provides some practice-based evidence that suggests that comprehensive family-driven SOCs appear to be associated with improved emotional and behavioral functioning over time among young children eligible for Medicaid in the San Francisco Bay Area’s Alameda County. Oftentimes, those with the lowest functioning need the most services over the longest period of time, and so that we still see a positive net impact of increased service participation on functioning measures that practitioners and families value is particularly promising. We recommend other counties implement similar systems to promote early childhood well-being.

Our study adds to the growing literature, including practice-based evidence, on the SOC approach. For agency-level practitioners, our results suggest county behavioral health care systems may benefit from using an integrated SOC approach such as that of Alameda County. Our findings also encourage working to support clients’ basic needs as well as their emotional and behavioral needs. We encourage service-level practitioners to pursue comprehensive SOCs for their early childhood clients, with an effort to engage families in continued service provision; our research suggests that this will lead to better child outcomes.

## Figures and Tables

**Table 1:** Child and family functioning in eight latent construct domains, for children birth-5 at service enrollment.

Latent Construct:	Cronbach’s α:	Items That Composed the Instrument:	Number of Children Observed	Prevalence (95%CI, Exact) of Construct
Outcomes of interest				
Early care and education	0.73	1. attendance 2. learning difficulties 3. group care interactions 4. conduct behavior	*n =* 488	56.1% (51.6–60.6%)
Emotional and behavioral functioning	0.82	1. anxiety/fears 2. separation problems 3. poor boundaries 4. depression 5. withdrawn 6. suicidality 7. ability to regulate 8. impulse control 9. oppositional behavior 10. concentration/attention span 11. aggressive/assaultive 12. verbal abuse 13. self-injurious behavior 14. obsessive/compulsive behavior 15. sexualized behaviors 16. unusual thoughts/verbalizations	*n =* 480	97.3% (95.4–98.6%)
Developmental functioning	0.69	1. gross motor 2. fine motor 3. speech/language 4. cognitive 5. feeding 6. toileting 7. sleep	*n =* 488	80.7% (77.0–84.1%)
Other needs				
Health problems that affect development	0.48	1. medical 2. dental 3. vision 4. hearing 5. receiving consistent medical care	*n =* 494	27.5% (23.6–31.7%)
Basic needs	0.87	1. housing 2. clothing 3. transportation 4. food	*n =* 491	41.8% (37.3–46.3%)
Safety	0.61	1. exposure to community violence 2. exposure to domestic violence 3. neglect/abuse	*n =* 488	56.8% (52.2–61.2%)
Social relationship issues	0.73	1. interaction with adults 2. ability to seek help from adults 3. interaction with peers	*n =* 493	89.2% (86.2–91.8%)
Family functioning	0.71	1. family stability 2. parent/caregiver-child relationship 3. sibling relationship(s) 4. parent/caregiver mental health 5. parent/caregiver substance abuse	*n =* 489	95.7% (93.5–97.3%)

**Table 2:** Sample characteristics of children birth to 5 with social, emotional and behavioral concerns served by MediCal Mental Health Providers in 2010 (*n =* 496), in comparison to the general Alameda County birth-5 population.

	Unique CFE Clients in 2010 (*n =* 496)	CFE Clients in Longitudinal Dataset (*n =* 148)	Alameda County Population Aged Birth to 5 (*n =* 122,309)
Gender			
Girls	42.4%	38.4%	49.0%
Boys	57.6%	61.6%	51.0%
Race/ethnicity			
Black/African American	25.2%	27.2%	12%
Asian American/Pacific Islander	9.5%	8.8%	25%
White	13.3%	10.2%	25%
Latino/a/x	45.8%	45.6%	32%
Native American	0.4%	1.4%	0.2%
Other/unknown	5.9%	6.8%	5%
Language of choice for service provision			
English	57.8%	55.5%	
East Asian or Southeast Asian (6 languages)	4.05%	2.7%	
Spanish	36.5%	41.1%	
Other non-English (3+ languages)	1.6%	0.7%	

Alameda County data from Alameda County data from First 5 Situational Analysis for Strategic Planning (2008) and California Department of Finance (2009).

**Table 3:** Association between care-related covariates and emotional and behavioral functioning score using a random-effects maximum likelihood estimation regression among children with more than one recorded observation in 2010 (148 individuals, 313 observations).

	ß Coefficient	95% Confidence Interval
Intercept	7.99	5.54, 10.44
Length of time participating		
2nd data collection follow-up	−1.68	−2.48, −0.88
3rd data collection follow-up	−2.97	−4.42, −1.51
Covariates		
English speaker	0.95	−1.09, 2.99
Age	1.12	0.53, 1.72
White	2.34	−0.83, 5.51

## Data Availability

The data supporting the analysis and results presented in this paper were shared with the author team by the Alameda County Department of Behavioral Health Care Services.

## Appendix A

**Table A1:** Correlation table between latent constructs of interest (*n =* 448 unique individuals in the dataset).

	ECE	EBF	Devel	Health	Basic Needs	Safety	Social Relationship	Family
Early care and education (ECE)	1.00							
Emotional and behavioral functioning (EBF)	0.53	1.00						
Developmental functioning (Devel)	0.40	0.41	1.00					
Health problems that affect development (Health)	0.08	0.06	0.43	1.00				
Basic needs	0.18	0.16	0.31	0.17	1.00			
Safety	0.15	0.27	0.18	0.05	0.49	1.00		
Social relationship issues	0.51	0.64	0.37	0.10	0.14	0.18	1.00	
Family functioning	0.13	0.27	0.25	0.13	0.52	0.58	0.22	1.00

Note: This table reports on the correlations between variables for each individual in the dataset at their first point of observation in 2010.
